# Diffuse infantile hepatic hemangioma successfully treated with propranolol orally: a case report and literature review

**DOI:** 10.3389/fonc.2024.1336742

**Published:** 2024-01-29

**Authors:** Zengyan Li, Zhiming Wu, Youhong Dong, Xiaojun Yuan, Dongdong Zhang

**Affiliations:** ^1^ Department of Oncology, Xiangyang No. 1 People’s Hospital, Hubei University of Medicine, Xiangyang, China; ^2^ Department of Orthopedics, Xiangyang No. 1 People’s Hospital, Hubei University of Medicine, Xiangyang, China; ^3^ Xinhua Hospital Affiliated to Shanghai Jiao Tong University School of Medicine, Shanghai, China

**Keywords:** infantile hepatic hemangioma, diffuse infantile hepatic hemangioma, propranolol, safety, efficacy

## Abstract

**Background:**

Infantile hepatic hemangioma (IHH) is a common vascular, fast-growing hepatic tumor that is usually accompanied by multiple cutaneous hemangiomas. Diffuse IHH (DIHH) is a rare type of IHH that exhibits many tumors with nearly complete hepatic parenchymal replacement. At present, there is no specific standardized treatment plan for DIHH. Herein, we present the case of a 2-month-old girl with DIHH and without cutaneous hemangioma who achieved complete remission after undergoing propranolol monotherapy.

**Case presentation:**

The infant with low birth weight was presented to the pediatric department with a 2-month history of persistent vomiting and feeding difficulty. Ultrasonography and abdominal magnetic resonance imaging revealed hepatomegaly and diffused intrahepatic lesions. A computed tomography-guided percutaneous liver biopsy was performed, and the pathological examination suggested the diagnosis was DIHH. The patient exhibited remarkably response to an increasing dose of oral propranolol, from 0.5 mg/kg to 2 mg/kg every day. The intrahepatic lesions were almost completely regressed after one year of treatment and no distinct adverse reaction was observed.

**Conclusion:**

DIHH can induce life-threatening complications that require prompt interventions. Propranolol monotherapy can be an effective and safe first-line treatment strategy for DIHH.

## Introduction

Infantile hepatic hemangioma (IHH) is a common benign mesenchymal tumor of the liver in fetuses and infants with a slightly high predominance in females. Approximately 90% of IHH cases are diagnosed within the age of six months and 30% are detected within the first month of life (1).

IHH was first described in 1919, and was frequently identified due to variable clinical symptomatology. The earliest known case of IHH were categorized into two subtypes in 1971 based on the tumor size and vascularity, namely type 1 and type 2 (2). IHH type 1 is usually considered benign and is the most common variant, whereas type 2 accounts for an insignificant portion of IHHs and can be malignant (3). In 2004, IHH was categorized into symptomatic and asymptomatic types based on the tumor size and hemodynamic effects (4).

A systematic classification scheme for IHH was introduced in 2007, which divided IHH lesions into focal, multifocal, and diffuse based on the clinical presentation, radiographic appearance, pathological features, physiological behavior, and natural history of patients (5). Focal IHH is generally manifested as a well-defined, solitary, and spherical tumor in magnetic resonance imaging (MRI). Most focal IHHs are asymptomatic. Only a few cases may present with minor anemia or thrombocytopenia, and cases are rarely accompanied by cutaneous hemangiomas. Multifocal IHH shared a similar MRI feature with multifocal lesions and may present with arteriovenous shunts. Nearly 60% of multifocal cases can present with more than five cutaneous hemangiomas (6) and some cases are associated with secondary high-output cardiac failure. DIHH presented as innumerable lesions spread into the entire liver, leading to the near-total replacement of the hepatic parenchyma. Infants with DIHH are more likely to develop serious complications, such as massive associated compression, congestive heart failure thrombocytopenia, and hypothyroidism, compared with those with the other two types (7).

Propranolol has been recommended as the first-line treatment for IHH in the American Academy of Pediatrics Clinical Practice Guideline (8). However, to date, there is no gold standard treatment available for DIHH. The efficacy of propranolol as a first-line treatment for life-threatening DIHH has not been demonstrated. Additionally, the optimal treatment choice for patients who do not respond to initial therapy with propranolol remains unclear. In this case study, we present a successful treatment of infant DIHH using conservative oral propranolol monotherapy. Based on our experience and the existing literature, we also propose diagnosis and treatment recommendations.

## Case presentation

A 2-month-year old infant girl with persistent vomiting and feeding difficulty was admitted to Xiangyang No. 1 People’s Hospital. The birth, family, and medical history of the patient were normal. The birthweight of the patient was 1.3 kg and she gained only 1.2 kg after 3 months. No cutaneous strawberry hemangioma was observed. The physical examination revealed that the liver could be easily palpated at 1 cm below the right costal margin. The laboratory tests, including routine blood tests, tumor markers, and thyroid function tests, including serum thyroid-stimulating hormone, free triiodothyronine, and free thyroxine, were all normal. Biochemical tests revealed slight mild increases in alanine aminotransferase and aspartate aminotransferase, at the level of 77.5 IU/L and 131.35 IU/L (normal 0-40 IU/L), respectively.

**Figure 1:**
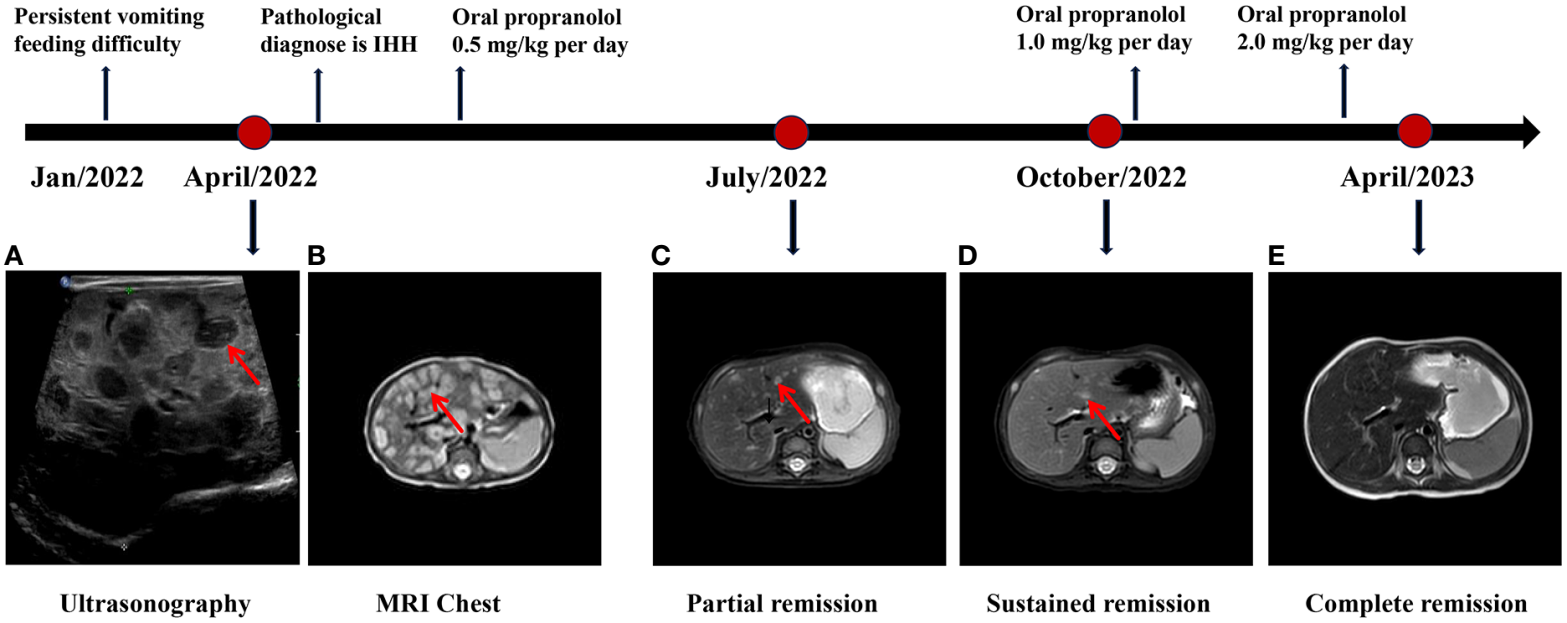
The diagnosis and treatment process of DIHH. **(A)** The abdominal ultrasonography indicated diffusely heterogeneous hepatomegaly with diffused rounded hypoechoic lesions. **(B)** The MRI revealed diffuse lesions with hyperintense pattern on the T2-weighted images in the liver. **(C-E)** The MRI indicates a continuous regression in the lesions during the 6 months follow-up, and ultimately reaching complete remission (CR).

**Figure 2:**
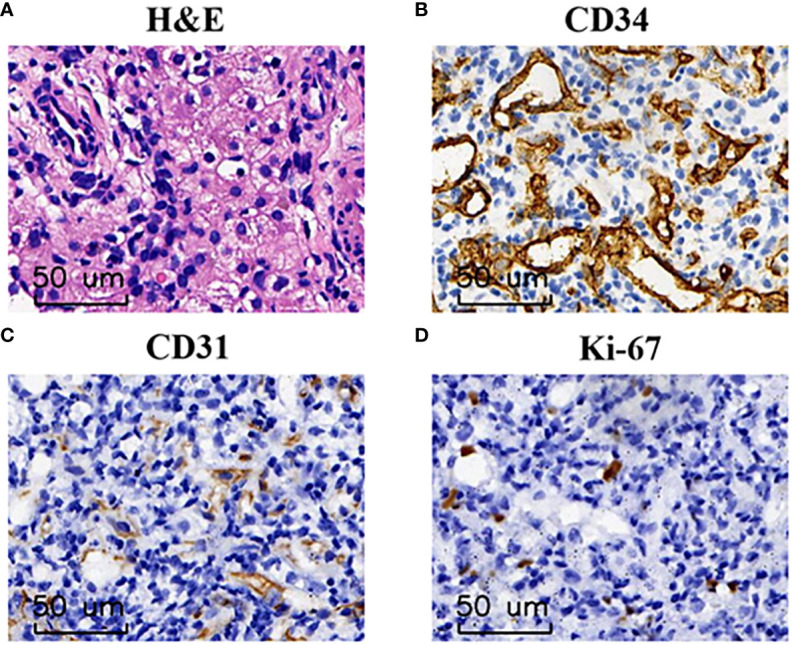
The pathological finding of DIHH. **(A)** Hematoxylin and eosin staining identified a large number of proliferating vascular endothelial cells at 200× magnification. **(B–D)** Immunohistochemical analysis indicated that tumor cells were immunoreactive for CD34 **(B)** and CD31 **(C)**, low Ki-67 index **(D)** at the magnification of ×200.

Abdominal ultrasonography (US) indicated diffusely heterogeneous hepatomegaly with diffused rounded hypoechoic lesions (**Figure 1A**). Cardiac ultrasound indicated mild-moderate mitral and tricuspid regurgitation and left ventricular ejection fractions was 60%. The MRI revealed diffuse lesions with the hypointense pattern on the T1-weighted images and hyperintense pattern on the T2-weighted images in the liver (**Figure 1B**). Accordingly, a computed tomography-guided percutaneous live biopsy was performed after the oral administration of chloral hydrate (0.5 ml/kg). Hematoxylin and eosin (HE) staining revealed that the tumor was composed of several proliferating vascular endothelial cells (**Figure 2A** **).** Immunohistochemical analysis showed positive staining for CD31 and CD34 (**Figures 2B, C**), with a low Ki-67 index (**Figure 2D**). Thus, the definite diagnosis was DIHH based on the imaging and pathological evaluations.

The patient underwent an oral monotherapy of 0.5 mg/kg of propranolol twice daily, which was gradually increased to 2 mg/kg/day. During the initial three days of treatment, the blood pressure, heart rate, and blood glucose levels of the patient were closely monitored and no reaction was observed. One week after the treatment, the vomiting symptoms of the infant stopped and milk consumption increase significantly. Most lesions were significantly regressed, and some lesions shrunk after three months of treatment (**Figure 1C**). The patient was persistently responsible for the current treatment and achieved near-complete remission after six months of treatment (**Figure 1D**). The intrahepatic lesions were completely regressed after one year of treatment and the patient was developing well (**Figure 1E**). The detailed diagnosis and treatment process is shown in **Figure 1**.

## Discussion

IHH is the most common pediatric vascular tumor in infants. IHH was previously called infantile hepatic hemangioendothelioma. The International Society for the Study of Vascular Anomalies first adopted the name IHH in 1982 based on the classification of vascular anomalies (9). IHH is commonly used instead of infantile hepatic hemangioendothelioma to avoid confusion with other malignant vascular tumors including Kaposiform hemangioendothelioma and epithelioid hemangioendothelioma.

IHH exhibits a slightly higher predominance in females than in males and familial tendency but without racial predilection (6). The reported incidence of IHH was approximately 1/20,000 newborns. The true incidence rate is underestimated because some cases are asymptomatic and undiagnosed. Previous studies have indicated that multifocal IHH is the most common type of IHH, whereas DIHH only accounts for > 20% of all IHH cases (10). This could be because multifocal IHH exhibits a higher probability of accompanying cutaneous hemangiomas than the other two types, which can be easily diagnosed (10).

The pathogenesis of IHH is poorly understood. One possible mechanism is the aberrant response of pluripotent stem cells to stimuli, including hypoxia and the renin–angiotensin–system. Focal IHHs are clinically and biologically distinct from multifocal and DIHHs (10). Multifocal IHH and DIHH manifest in the initial days and weeks of life. However, focal IHH is usually diagnosed prenatally. Moreover, all multifocal and DIHHs positively express glucose transporter isoform 1 (GLUT 1), which is a classic histological marker that differentiates IHH from other types of vascular anomalies. However, GLUT1 is rarely expressed in focal IHH (10, 11).

Approximately all cases of DIHHs are symptomatic and the clinical manifestations of DIHH are variable. The rapidly-proliferating masses can compress of the inferior vena cava, leading to hepatomegaly or abdominal distention, secondary hepatic failure, pulmonary embarrassment, and multi-organ system failure (12). These highly vascularized tumors can form arteriovenous shunts within the lesions, causing aggressive high blood volume and life-threatening congestive heart failure (13). The presence of several lesions can also trap of platelets within the tumors, resulting in anemia, thrombocytopenia, and consumptive coagulopathy (14). In addition, the overproduction of iodothyronine deiodinase type 3 and humoral thyrotropin-like factor by tumors is associated with hypothyroidism (15).

Generally, most cases of DIHHs can be diagnosed based on the clinical features and imagining results of the patient, and pathological examination is not required (16). US is the preferred imaging test for identifying DIHH, MRI can be chosen when the diagnosis is uncertain. Computed tomography is not recommended because it has a decreased resolution and an increased risk of radiation exposure in infants. A biopsy can be performed when clinical and imaging presentations are not atypical. In this case, we finally performed a biopsy using a fine needle aspiration to confirm the diagnosis due to the atypical clinical presentation.

DIHH can present with life-threatening complications and sporadic cases can undergo malignancy (17). Therefore, nearly all DIHH cases require systemic treatment. Several different medical therapies, such as steroids, interferon, and even cytotoxic agents, have been used for treating tumors and associated secondary symptoms (18). Corticosteroids were previously considered the first-line therapy for DIHH (5). However, owing to the subsequent side effects, such as abnormal fat deposits, gastric irritation, osteoporosis, and infection risk, its long-term application has been limited. Furthermore, approximately 23.1% of infants can present steroid resistance (19). Interferons were previously used to shrink tumors when patients were unresponsive to steroids (20). However, recent guidelines no long recommend interferon therapy owing to the risk of serious complications, including spastic diplegia and motor developmental disturbances (21).

Propranolol, a nonselective antagonist of β-1 and β-2 adrenergic receptors, was serendipitously found to pose an anti-proliferative effect in IHH in 2008 (22). The exact underlying mechanisms of propranolol in promoting hemangioma involution are unclear. It may be due to pericyte-mediated vasoconstriction, reduction of the vascular endothelial and basic fibroblast growth factor, and the inactivation of the renin-angiotensin system (23). In 2014, propranolol was approved by the American Food and Drug Administration (FDA) for the systemic treatment of IHH. Since then, propranolol was considered an effective and safe drug managing of IHH and it has replaced steroids (6). Furthermore, some case series have also demonstrated the efficacy of monotherapy with propranolol in DIHH (24, 25).

The recommended initial dose of propranolol by the FDA is 0.6 mg/kg, twice daily, with a gradual increase over 2 weeks to a maintain a dose of 1.7 mg/kg twice daily (8). European expert consensus group recommended that the starting dose of propranolol should be 1.0 mg/kg per day and a targeted dose should be 2.0 to 3.0 mg/kg per day and divided into two or three doses (26). The dosage should be adjusted based on the condition of the patient, especially in patients with (posterior fossa malformations, hemangioma, arterial anomalies, coarctation of the aorta/cardiac defects, and eye abnormalities) and adverse reactions, such as sleep disturbance (8, 27).

Treatment should sustain for at least 6 months and maintain until 12 months of age. Rebound growth may occur in 10% to 25% of patients during the tapering period or after the withdrawal period, which can occur even 6 months after the completion of therapy (28, 29). A large multicenter retrospective cohort study reported that patients whose therapy was discontinued at <12 months of age, especially below 9 months, exhibited the greatest risk of rebound. The lowest risk of rebound is in patients whose treatment was discontinued between 12 and 15 months of age (28).

Surgical or radiological intervention should be promptly performed when life-threatening symptoms progress after medical treatment or malignancy was present. Hepatic artery embolization (HAE) or Hepatic artery ligation (HAL) is an effective alternative approach to decrease shunts and counteracts cardiac failure (30). Orthotopic liver transplantation may also be chosen for patients with DIHH because total hepatectomy cannot be sustained (6).

Previous studies have reported that multimodal strategies, including corticosteroids, vincristine or propranolol and levothyroxine, have successfully treated DIHH (31). In our case study, considering the infant did not exhibit significant hypothyroidism or heart failure, we selected a conservative treatment with propranolol monotherapy. The patient achieved complete remission after one year of treatment, and no adverse reaction was found. This case study indicates that oral propranolol is an effective and safe conservative treatment for DIHH. The limitation of our study is that it is a case report, we believe that larger clinical studies to further investigate the effectiveness of propranolol monotherapy are still needed.

**Figure 3:**
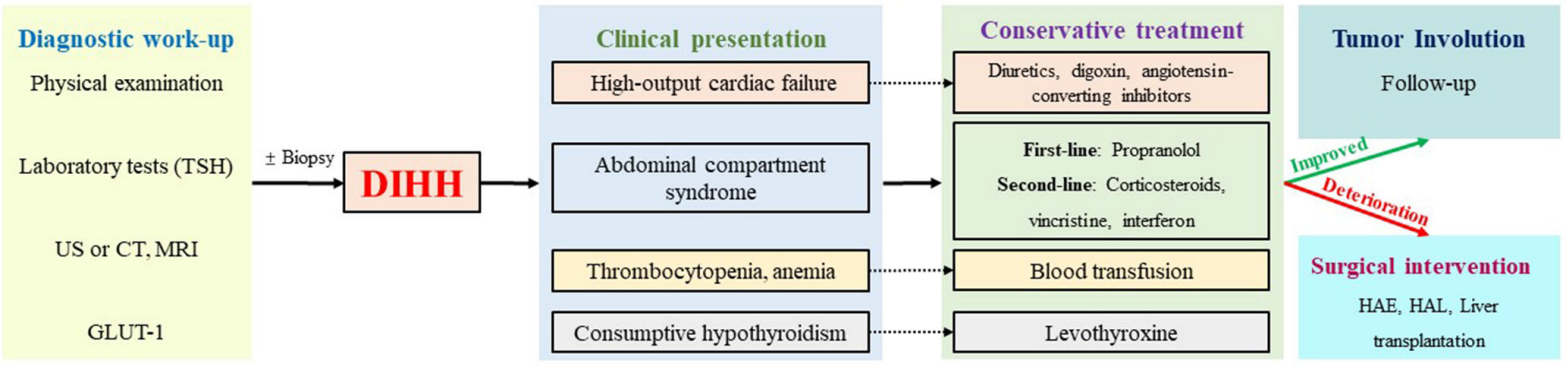
The diagnosis and treatment algorithms for DIHH. US, ultrasonography; CT, computed tomography MRI, magnetic resonance imaging; TSH, thyroid stimulating hormone; GLUT-1, glucose transporter isoform 1; HAE, Hepatic artery embolization; HAL, Hepatic artery ligation.

DIHH exhibits a relatively high mortality rate, with the reported highest rate of up to 53.8% (6). Serious complications, including abdominal compartment syndrome and heart failure, contribute to early mortality, whereas late mortality is mainly associated with incorrect treatment choices (32). Therefore, early diagnosis and prompt and effective intervention are important for treating DIHH. Based on the previous literature reports and our experience, we summarized the detailed diagnosis and treatment procedures of DIHH (**Figure 3**).

Propranolol can be recommended as first-line treatment for DIHH (33). In cases where patients are accompanied by high-output cardiac failure, diuretics, digoxin, and angiotensin-converting enzyme inhibitors may also be administered. Additionally, levothyroxine should be supplemented if consumptive hypothyroidism occurs, and blood component transfusion may be necessary in cases of hemocytopenia. For refractory cases, corticosteroids, vincristine, and interferon can be considered as second-line agents. In instances where pharmacologic therapy fails or there is deterioration, surgical intervention, such as HAE or HAL, was required to achieve satisfactory symptom control (14). In rare cases where the above treatments are ineffective, liver transplantation could be considered as an option (34).

To summarize, we have reported a patient with DIHH presenting having low body weight, persistent vomiting, and feeding difficulty. The patient did not have cutaneous hemangiomas or any other typical presentations and was completely cured after 1 year of oral propranolol monotherapy. We also propose an initial diagnosis and treatment algorithm for DIHH. We believe our study can provide some helpful insights into the precise diagnosis and treatment of DIHH in the future.

## Data availability statement

The original contributions presented in the study are included in the article/supplementary material. Further inquiries can be directed to the corresponding authors.

## Ethics statement

This study was approved by the Ethics and Scientific Committee of Hubei University of Medicine with approval number XYY2021002. Written informed consent was obtained from the individual for the publication of any potentially identifiable images included in this article.

## Author contributions

ZL: Data curation, Formal analysis, Methodology, Writing – original draft. ZW: Formal analysis, Methodology, Software, Writing – original draft, Writing – review & editing. YD: Data curation, Investigation, Project administration, Writing – review & editing. XY: Conceptualization, Data curation, Funding acquisition, Resources, Writing – review & editing. DZ: Conceptualization, Funding acquisition, Project administration, Writing – original draft, Writing – review & editing.
